# Hughes-Stovin syndrome revealing the presence of Behçet’s Disease

**DOI:** 10.22088/cjim.8.4.332

**Published:** 2017

**Authors:** Melek Kechida, Sondes Yaacoubi, Ahmed Zrig, Walid Jomaa, Rim Klii, Sonia Hammami, Ines Khochtali

**Affiliations:** 1 Department of Internal Medicine and Endocrinology, Fattouma Bourguiba Hospital, University of Monastir, Tunisia.; 2 Department of Radiology, Fattouma Bourguiba Hospital, University of Monastir, Tunisia.; 3 Department of Cardiology, Fattouma Bourguiba Hospital, University of Monastir, Tunisia.

**Keywords:** Behçet syndrome, Hughes-Stovin syndrome, Aortic aneurysm

## Abstract

**Background:**

Hughes-Stovin Syndrome (HSS) is a rare clinical disorder characterized by deep venous thrombosis and multiple pulmonary and/or bronchial aneurysms. Aneurysms in systemic circulation can also be seen.

**Case presentation:**

We report the first case of HSS with aortic aneurysm in a 55-year-old man who initially presented with deep venous thrombosis. The diagnosis of HSS revealing Behçet’s disease was made given the history of recurrent oral and genital ulcers. Treatment consisted of 3 daily pulses of methylprednisolone (1g) followed by oral prednisone (1mg/kg daily) and 6 monthly pulses of cyclophosphamide. Oral anticoagulation treatment was held 3 months and then was stopped with good outcome.

**Conclusion:**

Systemic aneurysms in Hughes Stovin is a worth knowing complication which may reveal Behçet’s disease.

Hughes-Stovin Syndrome (HSS) is a very rare clinical disorder characterized by deep venous thrombosis and multiple pulmonary and/or bronchial aneurysms. Aneurysms in the systemic circulation can also be seen. Given the scarcity of this disease, there are no defined descriptive criteria (1). HSS is diagnosed when deep venous thrombosis is associated to aneurysm, after ruling out other causes explaining such association (1). The pathogenesis of this syndrome is not known yet.. It is thought that this syndrome results from a vasculitis similar to the one implicated in Behçet’s disease (BD) which can have a systemic vessel involvement (2). In fact, histological studies showed destruction of the arterial wall and perivascular lympho-monocytic infiltration of capillaries and venules (3) similar to histological analysis in BD. Management of HSS is not standardized given the scarcity of the disease and is often treated like BD given similarities between the 2 syndromes. To the best of our knowledge, we report the first case of HSS with ascending aorta aneurysm.

## Case presentation

A 55-year-old man presented to the emergency room with pain and swelling of his left leg. Physical examination was unremarkable except edema of left leg and few folliculitis on the back. A color Doppler examination showed deep vein thrombosis. A chest x-ray revealed widening of the superior mediastinum associated with a history of chronic cough. Contrast-enhanced computed tomography (CT) showed an uncomplicated ascending thoracic aorta aneurysm of 48 mm (figures 1, 2). Echocardiography was normal. He gave no history of fever, hemoptysis or chest pain, but reported a history of recurrent oral and genital ulcers. 

Complete blood count, serum creatinine, erythrocyte sedimentation rate and urine analysis were within normal limits. Laboratory testing of associated thrombophilia revealed no abnormalities. Pathergy test and human leukocyte antigen (HLA B 51) were negative. The diagnosis of HSS revealing Behçet disease was made. Ophthalmic investigation revealed no uveitis or vasculitis. Treatment consisted of 3 daily pulses of methylprednisolone (1g) followed by oral prednisone (1mg/kg/j daily) and 6 monthly pulses of cyclophosphamide than azathioprine daily. Colchicine was associated too. Oral anticoagulation treatment was held 3 months and then was stopped with good outcome. A follow-up CT scan showed persistent ascending thoracic aorta aneurysm of 40 mm. 

## Discussion

To the best of our knowledge, we reported the first case, of Hughes-Stovin Syndrome (HSS) with ascending aorta aneurysm. HSS was first described in 1958 as recurrent thrombophlebitis of extremities and cerebral venous sinus associated with pulmonary aneurysms resulting in hemoptysis (1, 4). Then, other descriptions of HSS associated deep venous thrombosis with multiple pulmonary and/or bronchial aneurysms were reported (1). Few cases later were described with associated aneurysm of the systemic circulation such as bronchial, external carotid, iliac artery aneurysm and left hepatic artery (2, 5), but no association with ascending aorta aneurysm was reported. There are no formal diagnostic criteria for HSS. The diagnosis can be made if a patient presented with deep venous thrombosis and aneurysm after ruling out other differential diagnosis. Classically, HSS presents with hemoptisies secondary to rupture of pulmonary or bronchial artery aneurysms into the airways. 

In our patient, ascending aorta aneurysm was revealed by chronic cough with enlarged upper mediastinum in chest x-ray confirmed by CT scan. Deep venous thrombosis associated with ascending aorta aneurysm have made the diagnosis of HSS. BD is a recurrent systemic disease characterized by oral and genital aphthosis in association with neurologic, ophthalmic, gastro intestinal or articular involvement (6). 

Similarities can be described in BD given that the former can also be associated to thrombosis and aneurysms. Some authors believe that HSS represents a cardiovascular manifestation of BD. HSS has been variably described in literature as “the cardiovascular manifestation of Behçet’s disease”, “incomplete Behçet’s” disease and “a rare case of Behçet’s disease” (2, 3). Apart from that, histopathological findings show that HSS is characterized by destruction of the arterial wall and perivascular lympho-monocytic infiltration of capillaries and venules (3) similar to histological analysis in BD, which may show infiltration by lymphocytes, neutrophils and plasma cells in the media and adventitia and a proliferation of the vasa vasorum in the media as well as a fibroblastic proliferation (3). However, systemic involvements, like neurologic signs, mucocutaneous lesions, ocular and gastrointestinal manifestations are the hallmarks of BD and are symptoms which distinguish the latter from HSS.

Treatment of HSS is not standardized, given the fact that it is a rare disease. It is often tailored along the lines of BD, given the similarities between the 2 syndromes (5, 7). Treatment is based on glucocorticoids administered as pulse IV therapy followed by oral steroids with subsequent taper. In addition to that, immunosuppressor drugs are usually needed especially cyclophosphamide. Other immunosuppressors can be used like ciclosporine or azathioprine. The aim of the treatment was to stabilize small aneurysms and even make them regress in some cases like in our patient.

Anticoagulation remains a dilemma as it may prevent progression of thrombi but increases at the same time the risk and severity of hemorrhagic complications. In fact, it is usually contraindicated given the increased risk of fatal hemorrhage due to aneurysm rupture (5, 7) but can be employed with great vigilance in some cases with deep venous thrombosis associated with intracardiac thrombi of life threatening pulmonary embolism (5, 7). Surgical management is indicated in few cases with massive hemorrhage due to large pulmonary aneurysm consisting of lobectomy or pneumectomy (5, 7). For patients who are not suitable for aggressive surgical intervention, transcatheter arterial embolization can be indicated (5, 7). In our case, the patient received IV pulse therapy with corticosteroids of 1g/daily for 3 days instead of oral regimen with 1mg/kg/day associated with 6 pulses monthly of cyclophsphamide to a daily dose of azathioprine. Anticoagulation treatment with acenocoumarol was given for 3 months with periodic control of INR targeting 2. Afterwards, it stopped with a good outcome 1 year later. 

In conclusion, Hughes-Stovin Syndrome is a rare disease characterized by deep venous thrombosis associated with aneurysms in systemic circulation. It shares similar clinical, radiological and histopathological findings with Behçet’s disease. There are no standard treatment guidelines for the management of this disease which is generally treated like BD. Anticoagulation remains a dilemma associated in some patients according to the clinical presentation. 
